# Holocene vegetation patterns in southern Lithuania indicate astronomical forcing on the millennial and centennial time scales

**DOI:** 10.1038/s41598-019-51321-7

**Published:** 2019-10-11

**Authors:** Andrej Spiridonov, Lauras Balakauskas, Robertas Stankevič, Gražyna Kluczynska, Laura Gedminienė, Miglė Stančikaitė

**Affiliations:** 1 Department of Geology and Mineralogy, Faculty of Chemistry and Geosciences, Vilnius University, M. K. Čiurlionio 21/27, LT-03101 Vilnius, Lithuania; 2 Institute of Geology and Geography, Nature Research Centre, Akademijos Str. 2, LT-08412 Vilnius, Lithuania

**Keywords:** Palaeoclimate, Plant ecology

## Abstract

The Earth’s biota originated and developed to its current complex state through interacting with multilevel physical forcing of our planet’s climate and near and outer space phenomena. In the present study, we focus on the time scale of hundreds to thousands of years in the most recent time interval – the Holocene. Using a pollen paleocommunity dataset from southern Lithuania (Čepkeliai bog) and applying spectral analysis techniques, we tested this record for the presence of statistically significant cyclicities, which can be observed in past solar activity. The time series of non-metric multidimensional scaling (NMDS) scores, which in our case are assumed to reflect temperature variations, and Tsallis entropy-related community compositional diversity estimates q* revealed the presence of cycles on several time scales. The most consistent periodicities are characterized by periods lasting between 201 and 240 years, which is very close to the DeVries solar cycles (208 years). A shorter-term periodicity of 176 years was detected in the NMDS scores that can be putatively linked to the subharmonics of the Gleissberg solar cycle. In addition, periodicities of ≈3,760 and ≈1,880 years were found in both parameters. These periodic patterns could be explained either as originating as a harmonic nonlinear response to precession forcing, or as resulting from the long-term solar activity quasicycles that were reported in previous studies of solar activity proxies.

## Introduction

Solar activity patterns, which are mostly determined by the highly complicated magnetohydrodynamics in its interior, have a major influence on decadal to (at least) millennial variability in weather, climate and biota^1,2^. The primary evidence for the persistence of cyclic variation in solar activity over short time periods comes from direct satellite multispectral and visible light observations using telescopes^2–4^. Long-term observations of the Sun’s activity, including cyclic patterns, are mostly determined through studying fluctuations in cosmogenic ^10^Be isotopes and Δ^14^C isotopic ratios, whose production is regulated by the combined effects of the Earth’s and the Sun’s magnetic fields on low-to-moderate energy cosmic rays flux into the atmosphere^5,6^. Recently, nitrate ($N{O}_{3}^{-}$) concentrations in Antarctic glacier ice was suggested as an additional direct index of solar activity^7^. Analyses of these direct proxies, besides the very well-known 11-year cycles, have most frequently revealed the presence of so-called DeVries (~208-year) and Gleissberg (~80- to 90-year) cycles, as well as longer cycles that modulate shorter-term periodicities^8,9^.

The effects of solar forcing as determined from paleoclimatic and paleobiological proxies are multifaceted and variable in magnitude and effect type. Long-term solar activity was implicated in modulating the winter precipitation patterns, and thus the glacier expansion and contraction dynamics, during the Holocene in Alaska, northwest North America and the tropical Andes^10–13^. Strong support for the existence of 11-year solar cycles has been found in the recent foraminifera fossil record^14^. Solar cycles of different centennial frequencies have been detected in changes in the chemical composition (Mg/Ca ratio) of ostracode carapaces from the late Holocene on the Great Plains^15^. Ostracode community analyses have detected cyclical solar influence in the central European paleolake in the Panonnian Basin during the Miocene epoch^16^. Recent analyses have revealed that there is an entire hierarchy of solar cyclic forcing with predominant periods of 2,300-year (Hallstattzeit), 1000-year (Eddy), 500-year unnamed cycles, and 120-year unnamed cycles, along with the well-recognized 208-year (DeVries) cycles and 50- to 100-year (Gleissberg) quasi-cycles^7,17^. The longest solar cycles are claimed to have been detected even in the Devonian carbonate sequences from the Czech Republic^18^. Paleoecological peatland proxy records of climate change show consistent statistically significant peaks of periods lengths of 40 to 100 and 120 to 140 years, with a DeVries cyclicity (or Suess wiggle) of 200 to 210 years also being common, although sometimes spurious results could be expected due to autoregressive noise if false alarm levels for detecting periodicities are set to 90%^19^. Tree ring records are some of the most confident sources of short-term climatic (including solar) processes^20^, revealing that, possibly, ≈11-year (Schwabe) cycles were present and affecting plant growth at least from the early Permian period^21^, although controversy over this finding persists^22,23^.

Despite being subordinate to anthropogenic forcing, it is thought that solar influence has been a significant component of recent climate change, e.g. warming of the climate during the first half of the twentieth century^8^. Solar activity patterns in the past and their connection to the Earth’s climate and biotal response have led to some of the most challenging questions about the expected natural variability of the climate system^24,25^. Analyses of observational data on the climatic response show that there are significant regional effects in the amplitude and phase of response to cyclic solar forcing^2,26^. This observation points to the preeminent importance of studies on a regional scale, which could help us understand causally how relatively minor variations in solar insolation could produce major responses in climate and biota.

It is known that the climate system and its effects on the biota are characterized by several scaling regimes, with predictable (months to years; tens of thousands to hundreds of thousands of years) and highly unpredictable (minutes to months; decades to tens of thousands of years; hundreds of thousands to many millions of years) dynamical ranges^27^. This very broad range of time scales for the variability in biota and climate encompasses several well-documented connections to astronomical forcing. Extremely long-term periodicities on a time scale of tens of millions of years are likely to be connected to large-scale astronomical forcing through direct climatic effects (e.g. dimming of the Sun caused by encountering a molecular cloud), indirect climatic effects due to asteroid and comet impact effects or direct physiological effects due to increased radiation after supernovae explosions^28–36^. There is also growing evidence that shorter-term climatic perturbations related to the Milankovitch orbital mechanisms were of crucial importance in the ecological and macroevolutionary forcing of biotas during most of the Phanerozoic^37–42^. Although a connection between the Sun and the climate has been suggested and studied, starting from the earliest scientific studies^2^, there is no comprehensive understanding of the importance of solar forcing on the time scales of the Holocene epoch due to the unevenness of the geographical and temporal studies, and possible complexity in the geographical response of the climate and biota. Therefore, knowledge of the impact of solar activity, and other forms of solar insolation forcing in the “unpredictable” dynamic range between centuries and tens of thousands of years for the climate and biota affected by it, is crucial for predicting ecological and evolutionary dynamics on the Earth in the past, present and future.

In order to test the hypothesis of centennial and millennial solar forcing against a null hypothesis of random forcing with memory on the plant biota during the Holocene, a series of spectral analyses have been performed on the time series of pollen diversity and the compositional gradient scores of pollen assemblages. The studied data come from a coring site which was sampled in southern Lithuania (central Europe) in the Čepkeliai peat bog^43^, which has been virtually unaffected by human influence for its entire history and is currently a strict nature reserve, and is thus very suitable for studying natural variation.

### Site description

**Figure 1:**
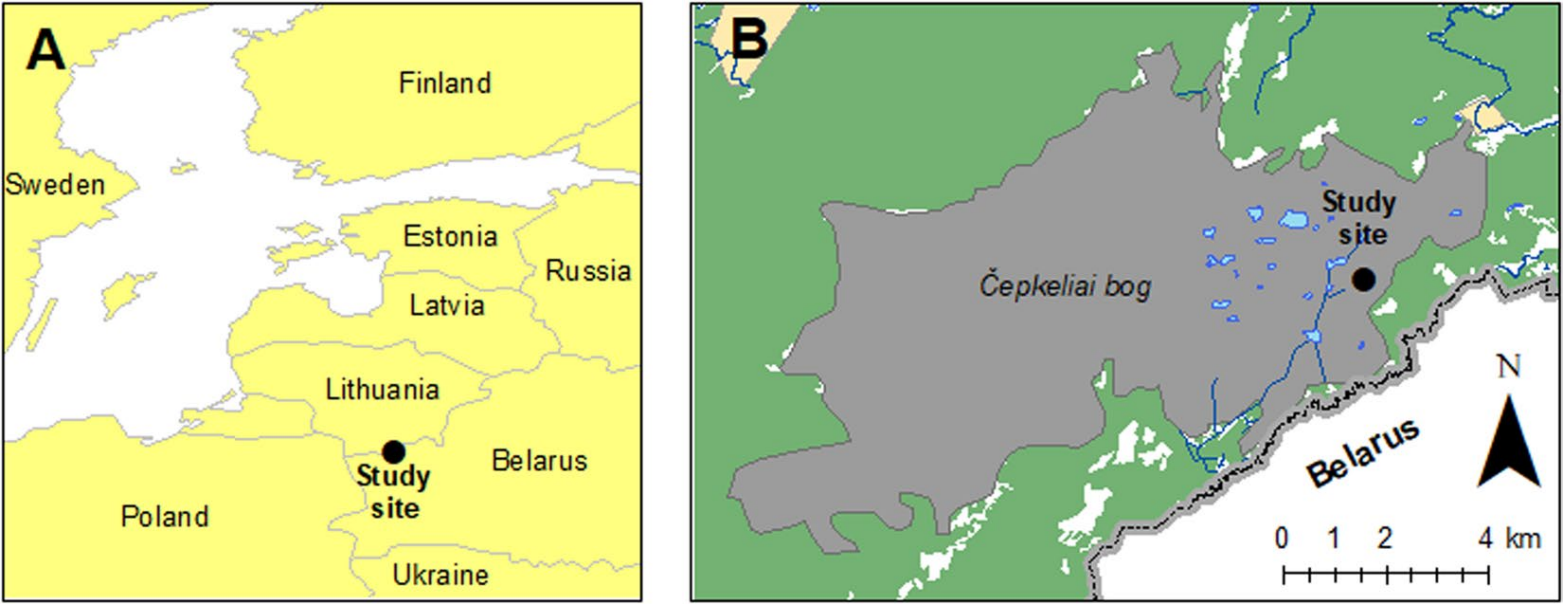
Geographical location of the core.

The Čepkeliai bog is part of a well-preserved, natural wetland complex (54°00′N; 24°30′E) covering an area of around 5,858 ha in south-eastern Lithuania and north-western Belarus (Fig. 1). Stretching along the marginal zone of the Late Weichselian ice sheet^44^ within the territory of the outwash plain^45^, the complex is bordered to the north by a massif of continental dunes from the Lateglacial age overgrown by dry pine forests^46^. In the north-east glaciofluvial uplands predominate, and end moraines on the Medininkai Highlands dating to the Middle Pleistocene adjoin to the north-east. *Sphagnetum magellanici*, *Ledo*-*Pinetum* and *Caricetum limosae* communities cover the surface of the wetland^47^. The mean annual precipitation fluctuates around 673 mm yr^−1^ mm, and the mean temperature is ~6.2 °C in July and −5.4 °C in January. There is snow cover for around 75–80 days a year.

### Data

Five parallel sediment cores were collected from the south-eastern part of the Čepkeliai bog (54°00′48.54″N, 24°37′01.02″E, 131 m a.s.l.) using a traditional “Russian” corer (1-m long inner chamber, ø 5 cm chamber). The depth of the postglacial sediments, including those of biogenic and limnic origin, reached 1,600 cm below the surface of the vegetation cover there. The sediments were sub-sampled at a 2-cm and 4-cm resolution for pollen and ^14^C analysis, respectively. As the scope of this paper is concentrated on the Holocene, the reported data relate to the corresponding part of the sequence, respectively. The multi-proxy data representing the Early and Mid-Holocene were discussed in detail in^43^ and the Late Holocene part was reported in^48^.

All together, 149 sediment samples covering a 100–1,227 cm interval were analyzed by means of their pollen composition in the case of the Čepkeliai sequence. Due to the possible contamination of the material, the uppermost part of the sequence (0–100 cm) was rejected. Sub-samples of 3 cm^2^ represent every second 2 cm-thick sediment slice. Standard chemical procedures^49^ were used to prepare the samples, followed by the identification of the specimens^50–52^. The minimum value of the identified specimens was at least 500 pollen grains of terrestrial plants per sample.

To construct the age-depth model, 12 bulk samples were dated using the radiocarbon (^14^C) method. The dating was performed at the Laboratory of Nuclear Geophysics and Radioecology at the Nature Research Centre in Vilnius following the conventional methodology with the application of liquid scintillation counting (LSC) by Tri-Carb 3170TR/SL. The benzene synthesis and purification were performed according to^53–55^. To calibrate the ^14^C data to calendar ages (BP) and construct an age-depth model, the Bayesian statistical method of the OxCal^56^ program with the OxCal age-depth P-Sequence model^57^ applying the IntCal13 dataset^58^ in OxCal v4.2.4^59^ was applied. The depth vs. accumulation rate curve and age uncertainty dates are presented in^43^.

Improving the accuracy of the age-depth model and tying the radiocarbon dates to narrower intervals on the IntCal13 radiocarbon calibration dataset^58^ (Reimer *et al*., 2013) applied calculations reduced the model’s possible uncertainties^48^. Moreover, the biostratigraphical data obtained on the regional scale were taken into account when discussing the age-depth model in Čepkeliai^60^. The sediment accumulation rate was calculated at 1 cm-deep intervals from the age-depth model’s median values. Having been obtained considering the lithological boundaries and representing different sedimentological units, the ^14^C data result in concordant ages with regard to depth and the sediment lithology in Čepkeliai. Moreover, appearing in stratigraphic order, the ^14^C dates suggest the absence of an old-carbon input or hard water phenomenon, a sedimentation hiatus, or any large deviations in the sedimentation regime and accumulation rate in the main part of the sequence.

### Statistical procedures of community composition and diversity estimation

The palynological data from the Čepkeliai bog, which were described in full in a previous article^43^, were analyzed here from a multivariate compositional and spectral estimation perspective in order to test the presence and nature of cyclic forcing of plant communities in central Europe. It is well known that pollen data do not directly correspond to vegetation patterns, although the correspondence between palynological taxa and Linnaean organismal taxa is very high in moderately diverse temperate forested regions in Europe^61^. Pollen production could have been disrupted or, conversely, enhanced, depending on the climatic perturbations despite essentially very small changes in the composition of the primary plant communities^62^. This property could be seen as an advantage for palynological data sets, as, in this manner they could respond faster to external forcing, thus better reflecting processes with higher frequency constants and working on time scales of tens to hundreds of years. Although generational changes in tree taxa could take hundreds of years, reductions in their growth, changes in grazing and fire regimes, and, ultimately, their mortality due to significant climate change could occur in decades or even years^63,64^, which should leave detectable changes in the pollen composition.

At first, the palynological data were explored in the light of the hidden environmental gradients. A usual way to explore the gradients in a biotic composition is to use principal component analysis and similar ordination techniques. However, the co-occurrence structure of species which is modal (mostly unimodal)^65^ can significantly distort the multivariate representation of the data in PCA analyses, as these techniques assume that all the variables are linear response functions^66^, and a hidden gradient could appear not as a straight line as it should be, but as an arching or spiralling pattern^67^, which could make an interpretation of the sample ordination meaningless. In order to mitigate this bias, we used a so-called non-metric multidimensional scaling (NMDS) technique, which iteratively randomly re-arranges samples according to their similarity and the chosen metric, in order to achieve a maximally consistent gradient in (usually) two dimensions^66,68^. The NMDS analysis was performed in the R environment using a ‘vegan’ package^69,70^. Before the NMDS analysis, the pollen count data were square root transformed and standardized using Wisconsin double standardization. For the further non-metric multidimensional scaling analysis, Euclidean distance was used. The stress value, or the proportion of unexplained variance in the pollen data by the two NMDS axes, for the global solution is = 0.18. The extracted NMDS1 sample scores, which explained 42% of the variance in the data, were later used as a time calibrated input value in further time series analyses (the raw variable values for the samples and taxa are presented in the Supporting Information). The NMDS1 axis, as in other ecological ordination techniques (e.g. detrended correspondence analysis), represents the main environmental gradient of the community compositions^66,71^. As our sections span the end of the Pleistocene and most of the Holocene (except the last ~700 years), it is highly probable that the most significant changes in the composition of the plant communities, as expressed by their pollen, were driven by major changes in the average temperature and related climatic covariates. It has been shown in many studies that in the northern Europe plant community, composition was determined by persistent changes from a cold and wet climate to a dry and warm climate^72^. This is especially true for the sections representing the eastern Baltic, as they documented sharp changes from an arctic to a moderate climate over the course of 12,000 years^73–77^. For the purpose of testing this assumption, we compared a global reconstructed temperature stack^78^ with the NMDS1 axis scores revealed here.

The described approach was complimented with a diversity estimation based on the entropy indices. For this purpose, we used the so-called q* (*q* star) index, which is based on the Tsallis entropy estimator (a generalization of the information entropy index) and is expressed in the following way:

1 $${H}_{q}\equiv k\frac{1-{\sum }_{i=1}^{W}{p}_{i}^{q}}{q-1}$$

Here, *W* is the number of microstates (i.e. taxa), *p*_*i*_ is the probability of the occurrence of a given state (pollen grain type) in an ensemble, *k* is the constant (usually equal to “1”) and *q* is the scaling constant of interest here^79^. It was shown that this family of estimators encompasses the whole set of traditionally used diversity measures as special cases^80^. It is mathematically equivalent to the so-called Hill’s numbers equivalents formula^61^, with the difference that parameter *q* can obtain not only integer values, but any positive values in the predefined interval (in our case it was (0; 5]). Furthermore, it was determined in previous studies that the number of rare species in a community is inversely related to the scaling exponent q*, which is the value of *q* that corresponds to the smallest value of Pielou’s evenness in an evenness vs. *q* graph^81^. In order to make this measure more intuitive (higher values mean greater diversity), we used its reciprocal (1/q*) in all further numerical analyses and graphical displays. These measures were calculated in an R statistical computational environment using the ‘vegan’ package^69,70^.

It is known without doubt that taxonomic diversity estimates (including those of fossil pollen) are directly related to the sampling intensity^61,82^. Therefore, biases in estimating trends and fluctuations depend on the sample size sufficiency^83^ and on the variance of the sample size. Our pollen data had average pollen counts equal to $\mu =598$, and $\sigma =110$. Therefore, we tested for a correlation between 1/q* and the sampling intensity. We found that there was a statistically significant but very low correlation between the counted pollen numbers and 1/q* (*r* = 0.24, $p(uncorr)=0.0023$). On the other hand, the correlation between the detrended 1/q*, or the residuals used in the spectral analyses, and the sample sizes was very low and statistically insignificant (*r* = 0.047, $p(uncorr)=0.56$). All these facts show that the sample size bias should be very insignificant in the current data set. Alternative data handling by estimating species richness using a standard rarefaction technique would dampen the true and biologically meaningful shorter-term/high-frequency variability (which is of interest here), as was revealed in previous studies^84^.

Peat bog sediments are not ideal for providing a very precise high-resolution chronology in comparison to, for instance, varved sediments, due to the well-known biogenic mixing effect. On the other hand, the simulations showed that temporal mixing induces increased autocorrelation and, therefore, smoothens the original signal^85^. Therefore, the observed fluctuations in the presence of time averaging are expected to be of higher significance (less likely to be produced by random fluctuations), representing longer-term states of communities than snapshot samples.

### Recurrence and joint recurrence analyses of pollen data

Recurrence plots are a nonlinear dynamics numerical analysis and visualization technique, which uses filtered distance matrices of ordered observations to detect patterns of similar states on all time scales^86^. This technique has been successfully applied for the detection, statistical testing and visualization of biotic events, change points, and spatial and temporal coherency in paleontological time series^87,88^. Moreover, it has been shown that recurrence patterns revealed by recurrence plots could be used as an accurate tool for characterizing climatic regimes in the Quaternary^89^. Therefore, recurrence plots are applied here as a way of visualizing the time series structures (trends, episodes of static composition and oscillations) of pollen assemblage change throughout the Holocene.

A recurrence plot is a filtered distance matrix which converts all the values in stratigraphically ordered points to black (if similar) or white (if different) points given some threshold level. Mathematically, the recurrence plot of compositional paleocommunity data is defined in the following way:

2 $${R}_{i,j}(\varepsilon )=\theta (\varepsilon -{d}_{ij}),\,{\rm{for}}\,i,j=1,\mathrm{...},N$$

Here, *R*_*i*,*j*_(*ε*) is the square recurrence matrix, given threshold dissimilarity *ε*, *θ* is the Heaviside step function which gives “1” if the difference is smaller than the predetermined threshold, and “0” in an opposite case, in this case *d*_*ij*_ = 1 − *C*_*ij*_, where *C*_*ij*_ is the Morisita-Horn similarity index between two compared assemblages^88^. The threshold value of the critical similarity level was tuned automatically in order to achieve the global recurrence rate RR = 30% (0.3 of all the points were classified as “similar”). We used a single threshold for a time series, thus highlighting the largest scale recurrence structures and the non-stationarities of the observed processes^89^.

Similarly, the joint recurrence plot, which compares the recurrence of two different time series of the same length and it, is defined as a product of two separate recurrence plots:

3 $$J{{R}^{\overrightarrow{x},\overrightarrow{y}}}_{i,j}({\varepsilon }^{\overrightarrow{x}},{\varepsilon }^{\overrightarrow{y}})=\theta ({\varepsilon }^{\overrightarrow{x}}-\Vert {\overrightarrow{x}}_{i}-{\overrightarrow{x}}_{j}\Vert )\cdot \theta ({\varepsilon }^{\overrightarrow{y}}-\Vert {\overrightarrow{y}}_{i}-{\overrightarrow{y}}_{j}\Vert ),\,{\rm{for}}\,i,j=1,\mathrm{...},N$$

Here, $J{{R}^{\overrightarrow{x},\overrightarrow{y}}}_{i,j}({\varepsilon }^{\overrightarrow{x}},{\varepsilon }^{\overrightarrow{y}})$ is the square matrix of the joint recurrence plot, ${\varepsilon }^{\overrightarrow{x}}$ is the filtering threshold of variable *x*, ${\varepsilon }^{\overrightarrow{y}}$ is the filtering threshold of variable *y*, $\Vert {\overrightarrow{x}}_{i}-{\overrightarrow{x}}_{j}\Vert$ and $\Vert {\overrightarrow{y}}_{i}-{\overrightarrow{y}}_{j}\Vert$ are the Euclidean distances between two points in time for different variables *x* and *y*^86,88,90^. The joint recurrence plot was constructed for the purpose of detecting similar fluctuations in the time series of NMDS1 (community gradients) and 1/q* (community diversities) during the Holocene. The recurrence rates of the separate recurrence plots of NMDS1 and 1/q* were selected to be equal to RR = 30%. This approach is useful when comparing simultaneous dynamics in two time series composed of different variables, which are directly incommensurable^86^.

### Spectral analysis methods

In order to test the time series for the presence of statistically significant periodicities, we performed a so-called REDFIT spectral analysis, which can handle unevenly sampled data and estimate so-called false alarm levels for the data which are characterized by red noise-like patterns, where long-term fluctuations dominate the record^91^. The REDFIT method estimates the spectrum from the unevenly spaced data using the so-called Lomb-Scargle Fourier transform^92^ with Welch overlapped segment averaging (WOSA), which splits the time series into a determined number of overlapping (by 50%) segments in which the spectra are determined independently, and which later averages these spectra for the determination of the final spectral solution^91^. In this application, we split the time series into three such segments. After the estimation of the spectrogram was complete, the pattern was tested against a stochastic (thus intrinsically non-periodic) autoregressive AR(1) model or, to be more precise, against a spectrum which was estimated from a parametrized AR(1) model. The latter mathematical function served as a null model for the autocorrelated (“reddened”) time series stochastic noise estimation. Red noise is a typical kind of noise in long-term geological data^93^ which can generate incoherent long-term oscillations by chance^94^. The estimation of the spectrum was performed using the PAST program^95^. The time series of the ecological estimates were detrended before the spectral analysis, as all of them exhibited strong first-order trends. The time series of the pollen samples’ NMDS1 scores were detrended using a second-order orthogonal polynomial function, thus cancelling out the parabolic trend, and the time series of 1/q* was detrended using a fourth-order orthogonal polynomial equation. The average resolution (time span between samples) was $\bar{\Delta }=76.19$ years; therefore, the shortest periodicity that could be determined without a distortion in the present data was 153 years. As the length of the observation window was ~11,000 years, we only analyzed those cycles which had at least two oscillations per studied time interval, thus a period lasting <5,500 years.

Complementary to the described approach is so-called continuous wavelet analysis – a time evolutionary spectral analysis method that allows periodic signals to be detected in both the time and frequency (period length) domains. For the purpose of localizing possible temporary periodicities we used the so-called Morlet mother wavelet. This enables the fairly accurate detection of periodic (sine-like) wave patterns in time as well as frequency space^42,96^. Before the analysis, we interpolated the detrended time series to equal 25-year time intervals. We discounted any features in the produced scalograms which had period lengths less than 153 years (the Nyquist limit^97^). The wavelet transforms were produced using a “biwavelet” package in the R computational environment^70,98^ using the scale normalization option, which mitigates the bias inherent in ordinary wavelets that down weights higher frequencies^99^. In addition to the wavelet analysis, using a Gaussian band-pass filter (parameter *a* = 2.5) implemented in the “astrochron” package^100^, we extracted the longest term periodicities detected in our study. The Gaussian filter does not distort phases of fluctuations^101^, which is useful for interpreting the timing and causality of events during the Holocene.

## Results

The NMDS1 values of individual pollen taxa suggest a strong climatic control over this variable. Thermophilous taxa tend to have positive NMDS1 values (*Fraxinus* 0.6, *Carpinus* 0.57, *Tilia* 0.52, *Quercus* 0.43 etc.), while taxa associated with colder climates (e.g. *Selaginella selaginoides* −1.22, *Betula* −0.26) have negative values. The NMSD1 values for most predominating herb species (Caryophyllaceae −0.53, Chenopodiaceae −0.32, Poaceae −0.28, *Artemisia* −0.28, *Filipendula* 0.26, Cyperaceae −0.17 etc.) and shrubs (*Juniperus* −0.69, *Salix* −0.58) are also negative. The above-mentioned herb and shrub pollen taxa should instead be considered indicators of open areas than of cold climates as such. However, they can indicate colder stages indirectly, as increased landscape openness is characteristic of both the beginning of the Holocene and recent millenia^48^. *Pinus* (−0.17) and *Picea* (0.11), which were among the dominant taxa throughout the Holocene in the study area (Balakauskas, 2012), have low NMDS1 values. Many representatives of a limnic or telmatic environment (*Typha* −0.01, *Potamogeton* 0.02, *Nuphar* 0.04), including spores (*Sphagnum* −0.07, Polypodiaceae −0.05), have NMDS1 values close to zero, i.e. the abundance of their pollen is less related to climate change.

**Figure 2:**
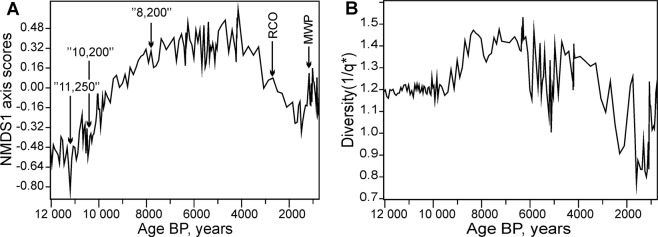
(**A**) Trend in the NMDS1 scores; (**B**) Trend in 1/q* of the fossil pollen assemblages. RCO – Roman Climatic Optimum, MWP – Medieval Warm Period.

**Figure 3:**
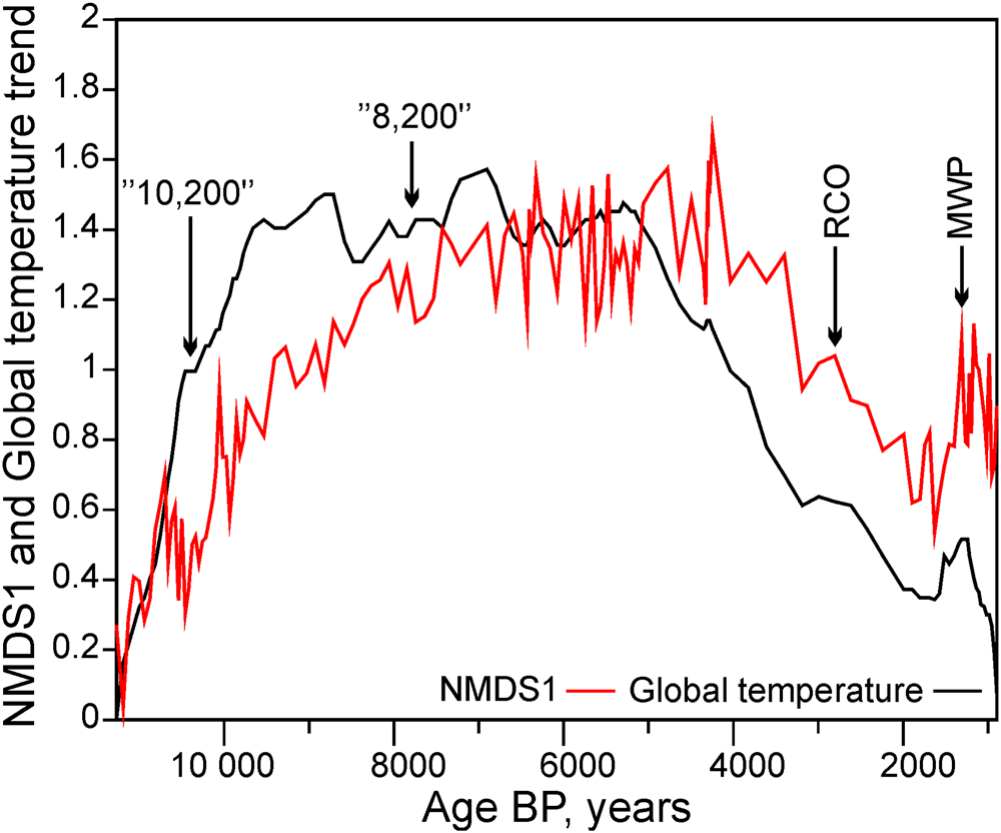
Comparison of the standardized NMDS1 trend and the standardized global temperature stack trend^78^ during the overlapping time interval (11,285 to 762 cal BP years). The standardization was produced by adding to the time series its minimal value and then dividing it by its average. RCO – Roman Climatic Optimum, MWP – Medieval Warm Period.

A visual inspection of the NMDS1 scores and 1/q* value plots (Fig. 2) shows that in the period between 8,000 cal yr BP and 3,300 cal yr BP there was a time period of high diversity which was, most probably, associated with a period of relatively stable and high global temperatures (Fig. 3) – the so-called Holocene thermal maximum, well expressed in eastern Baltic areas^78,102^ and dating back to 8,000–4,500 cal yr BP here^103^.

In our data, the NMDS1 scores show a relatively delayed response on the trend scale in comparison to the global temperature curve. Nevertheless, shorter-scale features, such as the so-called Preboreal Oscillation^104^ centred around 11,250 cal BP, the “10.2 ka” climatic oscillation^105^ and the putative “8.2” climatic event (although the latter one seems to have been delayed at the site under study^43^) were detected. “The Roman Climatic Optimum” and the “Medieval Warm Period” (Fig. 3) can also be directly distinguished and compared to the global surface temperature anomaly stack^78^.

**Figure 4:**
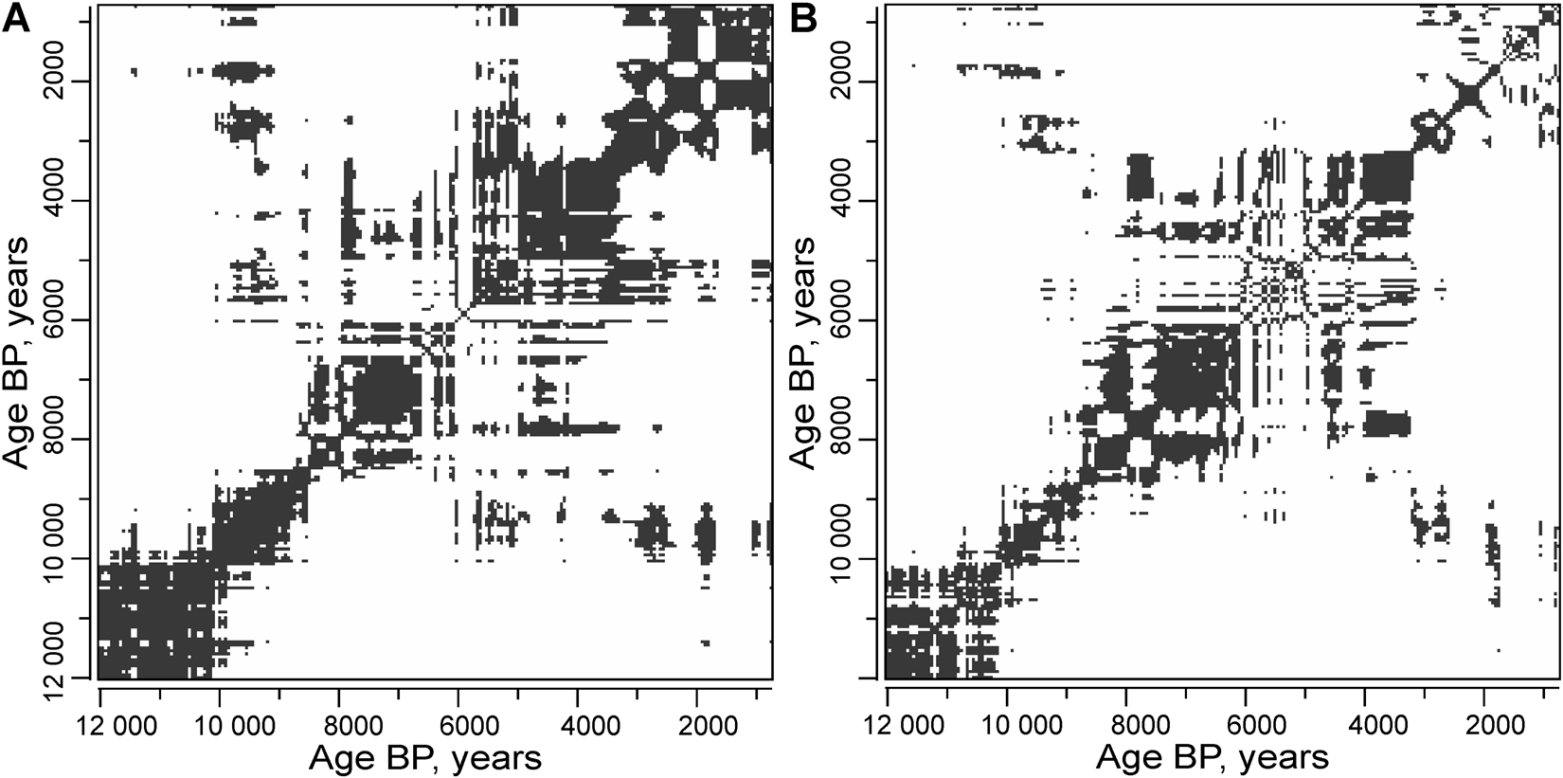
(**A**) Recurrence plot of the pollen taxa in the Čepkeliai section (Recurrence Rate = 30%); (**B**) Joint recurrence plot between the NMDS1 and 1/q* time series (Joint Recurrence Rate = 13.2%). All the time series were interpolated to equal 50-year time intervals.

A visual inspection of the recurrence plot of the pollen taxa composition (Fig. 4A) shows that there was a persistent trend in composition (the recurrence is nearly diagonal) from 12,000 yr BP to 8,000 yr BP. From that point, a long and stable state started to prevail (the recurrence is far from diagonal), which was later changed by the trend towards the modern distinct phase, which was partially recurring with the early Holocene taxonomic composition, indicating that during the period from 3,300 cal yr BP to 762 cal yr BP there was a transition to a colder state. More details can be seen in the joint recurrence between the NMDS1 and 1/q* time series, which are similar in exactly the same fluctuations between these two variables (Fig. 4B). The plot’s most significant feature is a very large region of joint recurrence in the middle Holocene, when both (presumably) the temperatures and pollen diversity were relative stable during the period between ~8,000 and 3,300 cal yr BP. However, there is a notable feature – a marked disruption in this pattern (a white cross-like structure) in the overall highly recurrent (stable) middle Holocene during the period between 6,000 and 4,000 cal yr BP. This feature indicates an increase in variance and a lack of overall recurrence in the shorter-term (centennial to multi-centennial) fluctuations.

**Figure 5:**
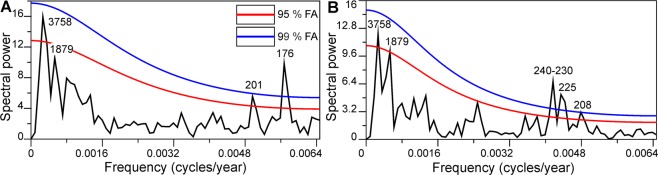
(**A**) Spectrogram of NMDS1; (**B**) Spectrogram of the 1/q* values. FA – 95% and 99% false alarm levels for statistically spurious spectral peaks.

A spectral analysis of the NMDS1 axis scores shows that there is a range of statistically significant cyclicities (Fig. 5A). Cycles lasting 3,758 and 201 years pass the 95% false alarm level. Cycles of 176 years are very distinct from the background autoregressive noise and pass the 99% false alarm level. Similarly, a spectral analysis of the time series 1/q* residuals shows the same 3,758 periodicity, and cycles lasting 1,879 years pass the 95% false alarm level. In addition, there is a cluster of periodicities that range from 208 to 240 years that pass the 99% false alarm level. Interestingly, although the periodicity with the period length of 1,879 years did not pass the 95% level, it still forms a distinct peak in the power spectrum of the NMDS1 time series (Fig. 5A), which points to similarities in the oscillatory processes in the NMDS1 scores (sample positions on the major environmental gradient) and 1/q* (diversity) values on very long time scales.

**Figure 6:**
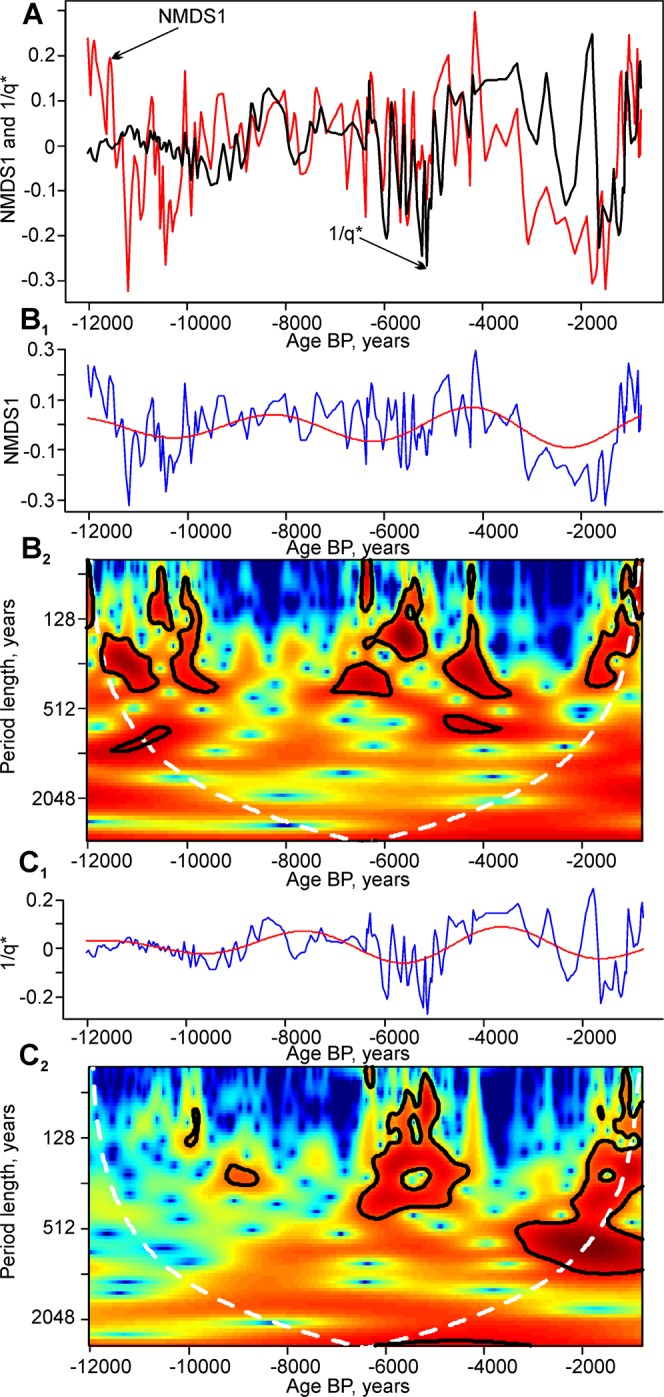
(**A**) Comparison of the detrended time series of NMDS1 (red) and 1/q* (black); (**B**_**1**_) Detrended time series of NMDS1 with band pass filtered longest cycles (≈3,760 years); (**B**_**2**_). Wavelet scalogram of NMDS1; (**C**_**1**_). Detrended time series of 1/q* with band pass filtered longest cycles (≈3,760 years); (**C**_**2**_). Wavelet scalogram of 1/q*. In (**B**_**2**_,**C**_**2**_), Nyquist limit of the cycles is 153 years.

A multiscale wavelet analysis of the detrended NMDS1 scores and 1/q* diversity indices revealed their time-specific periodic features (Fig. 6). A visual inspection of the detrended time series (A, B_1_, and C_1_) revealed close similarities in the phases and amplitudes of the longest detected periodicities of ≈3,760 years. An increase in the amplitudes of the longest fluctuations was documented, with long-term lows in the time intervals between 11,000 and 9,500 cal yr BP, 7,000 and 5,000 cal yr BP, and 3,000 and 1,000 cal yr BP. The records of the NMDS1 and 1/q* bicentennial cycles show marked differences (Fig. 6B_2_, C_2_). The NMDS1 time series appears to be more stationary with regard to short-term variance. Even though the ≈150 to 250-year cycles show amplitude modulations through time, they can be detected at the beginning, in the middle and at the end of the record. In contrast, the biodiversity (1/q*) time series shows a marked episodic increase in variance through time (Fig. 6C_1_,C_2_). The appearance of bicentennial cycles in this metric can be detected in the time interval between 6,000 and 4,000 cal yr BP, and between 2,000 and 700 cal yr BP. These bicentennial cycling episodes appear to be related to the lows of the long-term biodiversity cycles (Fig. 6C_1_). The high amplitude cycling (instability) episode in both NMDS1 and 1/q* between 6,000 and 4,000 cal yr BP can also be directly observed in the recurrence plots (Fig. 4A,B), thus confirming its significance.

## Discussion

The general trends in plant communities as revealed by the NMDS1 scores, the diversity index time series (1/q*), and the compositional recurrence and joint recurrence plots are highly consistent with the perceived picture of climatic evolution; low temperatures at the beginning of the Holocene, increasing temperatures throughout the Holocene Thermal Maximum and gradual cooling during the subsequent interval. A possible reason for the subtle delay in the NMDS1 trend compared to the global surface temperature stack, especially at the beginning of the record, is the gradual thawing of the permafrost at the start of the Holocene and the equilibration of the shallow geothermal field. The easterly influence^106^ on the climatic regime that predominated in this part of northern and north-eastern Europe before the final melting of the Scandinavian ice sheet between 10,000 and 9500 cal BP could be indicated as a moderating factor for the described situation. The frozen ground should have had significant temperature and groundwater effects on the possibility of the colonization of pioneering vegetation successions. The temperature attenuation time in any given geological section, besides the lithology and the hydrological conditions, depends on the magnitude and longevity of the climate change. Due to the soil’s significant thermal inertia, at depths of tens of meters warming could perpetuate after centuries of constant heating at the surface^107^. Other factors could be: the equilibration of communities after a transient period of wide dispersal following an ice age, which would depend on the species, and large-scale regional variations in the climate’s response to global orbital forcing, which is implicated in the late Holocene gradual cooling trend in the northern hemisphere. Abundant variations in the timing of response have been detected in proxy and model simulations^108^.

The longest ≈3,760-year period cycles (with a possible error of a couple of hundred years) in the NMDS1 scores and 1/q* values described in this study have analogues in widely distributed records. Cycles of comparable periods with adjustments for possible dating and spectral analysis uncertainties (3.4 to 3.6 ka) have been detected in the Quaternary sections of Lake Van in eastern Turkey^109^. Comparable cyclicities (4.4 to 3.7 ka) have been detected in the Quaternary foraminifera paleoecological data at different sites in the Indian Ocean, thought to be a result of the combination tones of Milankovitch (precession and obliquity terms) cycles^110^, which are an expected result of the external forcing of non-linear hydrological – glacially influenced systems^111^. Similarly, it has been found in modelling studies that sub-Milankovitch (<15 ka periods) cycles are expected in vegetation dynamics as a response to precession and obliquity-related perturbations^112^.

The discovered ≈1,880-year periodicity (a period length accurate to within a couple of hundred of years) is significant in diversity (1/q*) time series and, as has been revealed, is pronounced but insignificant in the NMDS1 time series. This contrasts to the dominant ~1.0 ka cycles that have been found in North American vegetation and lake sedimentation patterns in the Holocene records^113,114^ and the 1.2 to 1.5 ka cycles in Greenland and Atlantic paleoclimatic proxies^115,116^. These are probably more comparable to the somewhat longer 2.0 to 3.0 ka cycles (so-called “Bond cycles”) of the North Atlantic iceberg discharge^117^ and climatic fluctuations during the final stages of the Pleistocene and the beginning of the Holocene^118^. These so-called Bond events could not have been cycles in the strictest sense of the world, but approximately evenly recurring events or non-linear state shifts in the climate^119^. Cycles lasting 1,657 years have been estimated from sedimentation patterns in the lakes of central Finland^120^, which suggests similarities in the climatic forcing in the broader central to northern Europe region. Cycles of comparable duration (1,785 years) have been detected in *Globigerina bulloides* and the Rb/Sr ratio time series from the Late Pleistocene to the Holocene in the Indian Ocean^121^, and in molecular alkenon data from north-western Africa during Terminations II and IV, where cyclicities lasting 1,600 or 1,800 years have been found^122^. These cycles are thought to have originated from the non-linear effects of orbital forcing which resulted in higher frequency harmonic oscillations^122^. Similarly, the long-term periodicities of 3,846 years and 1,879 years appear to have been harmonically related (the first periodicity is almost exactly twice as long as the second one), they also appear to have been harmonically related (3,846 * 6 = 23,076; 1,879 * 12 = 22,548) to the modern Earth’s precession of equinoxes index (which is characterized by dominant longer periodicities of 23,600 and 22,326 years^123^). On the other hand, multiple cross-wavelet coherency analyses of solar proxies show that there were statistically distinct transient solar activity periodicities of ~1,885 years during the Holocene^7^. The same team of authors report statistically significant periodicities (1,585 and 3,358-year periods) comparable to ours, with close scales and period length ratios in the nitrate concentration solar activity proxy in the East Antarctic ice sequences^7^. Conversely, current analysis suggests that long-term solar cycles lasting ~500, 1000 or 2400 years, which are recognized in some paleorecords^16^, are not present here, or at least are not distinct enough against the background noise.

The higher frequency cycles of 201 to 240 years, which were detected in our study, could be connected more confidently to solar activity patterns. Cycles of very similar duration with spectral peaks of 199.4, 208.4 and 230.6 years have been detected in the Holocene δ^14^C time series^93^. Spectral analyses of the northern hemisphere temperature records from the last two thousand years have also found statistically significant cyclicities of 250 years^124^, close to the DeVries solar cycles. Fossil records of the tree coverage in Ireland show similar cyclic variations on the scale of 209 to 230 years, although these fluctuations are not in phase with solar forcing, which indicates the presence of nonlinear response mechanisms^125^. The Holocene record of biogenic silica and the diatom community structure in British Columbia has revealed that there are cycles of 241 to 243 years that are coherent with solar activity fluctuations^126^. Recent data on the Indian summer monsoon proxies have revealed statistically significant oscillations on a 230-year frequency band^127^.

**Figure 7:**
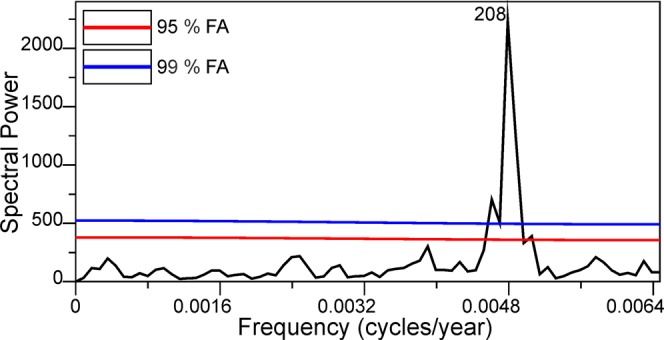
Spectrogram of simulated and summed 208 and 85-year cycles of equal amplitude for the presence of aliasing effects given the sampling properties of the Čepkeliai section. FA – 95% and 99% false alarm levels for statistically spurious spectral peaks.

The most prominent shorter-term cyclicity (176) detected in our data (NMDS1 scores) is almost precisely equal to two Gleissberg cycles or to a full Jose cycle (178.7 years)^128^. It was shown in earlier studies of solar activity/cosmic ray intensity proxies’ spectral analyses, that the so-called Gleissberg cycles, lasting approximately 87 years, are also very prominent in multiples of 4, 6 and 8^9^. There could be two principal explanations for the existence of this cyclicity in our record: a) aliasing and b) subharmonic forcing. The first explanation of aliasing indicates that as the sampling was performed at a lower frequency than the Gleissberg cycles, the aliased frequency could be an integer multiple of the original frequency^129^. On the other hand, variable (in the time domain) sampling, as is the case in this contribution, significantly diminishes the possible effects of aliasing^92,129^. We tested this possibility using simulation results. We generated two periodic processes that were equal in amplitude and with period lengths of 208 years (DeVries cycle) and 85 years (Gleissberg cycle) with durations equal to those observed in our record; later on we sampled at exactly the same pollen sampling points in the Čepkeliai section, thus mimicking the sampling features of our study. What is obvious from this experiment is that the variance from the 85-year periodicity should be completely dispersed throughout the very wide spectral range, and that it will not appear as a significant lower frequency peak in the vicinity of the 208-year periodicity (Fig. 7). On this ground we reject this possibility. It could be argued that the revealed ~240 to ~176 cyclicities are fairly close to the limits of the resolution and that their detection and significance could be problematic; the direct simulation which mimics the sampling unevenness clearly shows that it is sufficient in order to detect these short cycles with a high degree of accuracy. The dating uncertainties should affect shorter cycles the most by leaking spectral power to the neighboring frequencies. Luckily, this bias works toward accepting a null hypothesis of the absence of cycles, as the energy from a singular powerful peak would be spread throughout the range of lower and higher frequencies, thus diminishing the probability of accepting a distinct periodic process. Moreover, the dating errors would induce a phase distortion in the periodic oscillation, which would work against the recognition of persistent periodicities by cancelling the oscillations out. All this reasoning supports the reality of the centennial oscillations that are detected in the present study.

The uncertainty of the age estimates at the calibration points (68.2%) was around 250 years^43^. This would translate into an uncertainty of up to several hundred years per couple of thousand years when dealing with shorter-term fluctuations. This unaccounted variability in the absolute dating of samples should result in a possible short-term “frequency wandering” of a given centennial scale periodic signal with apparent cyclicities “condensing” or “stretching”. This possibly could result in artificial deviations of statistically significant centennial periodicities by up to several tens of years. But, as mentioned before, if these distortions had been very severe, we would have witnessed a significant dispersal of the spectral power throughout the range of frequencies (cycle lengths), which would have severely diminished their statistical significance (no coherent signal). What we observe here are relatively sharp peaks in the spectra of both the NMDS1 scores and 1/q*. The NMDS1 scores also show high persistence throughout the entire Holocene.

The uncertainties for longer (millennial) cycles should be larger if we are dealing with absolute time (up to 300 years), but much smaller on a relative scale. The overall uncertainties in the duration of the whole range (up to several hundred years) are far smaller than the probable duration of the studied interval (≈11,000 years).

Analyses of peatland sedimentary records have revealed the presence of cyclicities with differing period lengths (including bicentennial cycles). Internal self-organizing drivers linked to changes in vegetation occupancy were implicated in decadal to centennial cycles^130^. Centennial cycles lasting from 196 to 396 years were detected in the humification records of Swedish raised bogs^131^, although the cyclicities were connected to larger-scale climatic shifts that in turn were related to solar activity. Multivariate analyses of plant assemblages from the Walter Moss (England) peat record have also revealed the presence of cyclicities, although 200-year cycles were not detected, and the authors suggest a possible long-term averaging effect that mire sedimentological conditions could have had on the macrofossil data^132^. In our case, we used pollen data for the paleoclimatic and paleoecological analysis, which are representative of a much wider region than the bog itself. Therefore, autocyclicity is an unlikely reason for the short-term variations in the compositions of assemblages. On the other hand, bioturbations work toward a local homogenization of the fossil records (the low pass filtering hypothesis^132^). Therefore, the presence of significant and coherent short-term (centennial) fluctuations in pollen assemblages is an indication of the preservation of primary fine detailed records.

On the other hand, the previously discussed periodicity of 176 years could more probably have directly arisen through external solar forcing due to the non-linear effects (of a climate or vegetation response) of a forced system, which is dependent on the amplitude of forcing and other effects. This non-linear dependency could have resulted in subharmonic oscillations equal to *w*_*f*_/*k*, where *w*_*f*_ is the forcing frequency and *k* is an integer coefficient^110^. The latter option is more likely, as the forested biomes that surrounded our study site have long (decadal to centennial) characteristic response times to climate perturbations, which could have led to the observed oscillations. This encompasses a succession of communities which react with a delay, i.e. due to differences in seed masses^133^. This delayed response most possibly induced the non-linear period length amplification of the oscillations. A similar explanation was given for the late Holocene varve sedimentation patterns in Finland, where the authors also proposed that their 160-year periodicities were related to the harmonics of the primary Gleissberg cyclicity^134^.

Cycles 170 years in length have also been putatively detected in multispectral temperature records in a range of measurements between AD 1000 and 1935 in the northern hemisphere^124^. Tree ring data from the Colorado Plateau have yielded periodicities equal to 178 years in length^135^. A 177-year periodicity was detected in the Holocene *Pinus* percentage data from the eastern North America (Chesapeake Bay) region^136^. Periodicities of a comparable length of 187 years have been determined from the *δ*^13^*C* time series from the last two millennia, constructed from Japanese cedar tree ring data^137^. A Qinghai Lake (Tibet Plateau) sediment reflectance study revealed a 163-year periodicity in the Holocene strata^138^. A high-resolution Ti concentration record in Late Holocene sediments in eastern Finland has revealed a 160-year periodicity which was linked to solar activity^134^. Similar cyclicities (160 to 170 years in length) have been detected in the abundance time series of *Picea* in a high-resolution record of the paleolake in the Panonnian Basin during the Pliocene epoch, which were explained as heterodyne frequencies which appeared due to the interference of the Gleissberg and DeVries frequencies^139^. Finally, during the time interval between 8,000 and 4,000 cal yr BP, dry episodes with recurrence times of 160 ± 40 years have been detected in the Chew Bahir Basin sediments from southern Ethiopia^89^, which indicates the possible wider geographical persistence of this dynamic pattern during the Holocene.

It is thought that Gleissberg cycles are very persistent on the scale of almost the past ten thousand years^9^ (and probably the entire Holocene^140^ or even longer up to the Miocene^139^) of all the detected cyclicities in solar activity proxies, as they have been detected in a number of marine and terrestrial proxies^141–143^. Gleissberg cycles, as shown in previous studies, are also probably related to the gravitational perturbation effects of Jovian planets on the solar dynamo^144^, which could explain their persistence and regularity in the paleoclimatic record.

One of the most probable proximate mechanisms for the influence of variations in solar activity on the climate is their effect on meridional temperature patterns^136^. Theoretical and observational studies have revealed that the solar wind is directly responsible for the modulation of the global electrical circuit through modulating lower energy galactic cosmic rays, which affects the persistence of clouds (especially those formed over marine territories) through the changes in the formation of ice particles and their evaporation, which in turn affects changes in the albedo and intensity of winter cyclones^145,146^. This microphysics-based scenario is supported by the observation that charged embryonic water vapor condensation clusters are much more stable and persistent compared to neutral clusters, due to the Coulomb attraction force, thus making precipitation more likely^5^. As the climate, and precipitation in particular, in the Baltic region is directly influenced by the Atlantic Gulf Stream nowadays^147^, it is highly probable that this kind of coupled solar-oceanic forcing was a significant factor which modulated the temperature and precipitation patterns in the given region during the previous intervals of the Holocene as well, or during the middle and late parts of the interval in particular. The correlation between geomagnetic activity and particle fluxes with winter temperature anomalies during the descending phases of solar activity in the northern hemisphere, supports the hypothesis of the indirect solar forcing of climate through the modulation of electromagnetic microphysics phenomena in the atmosphere^148^.

Another source of climatic influence of solar variability is the increased UV absorption by the ozone and the heating of the stratosphere, which disturbs the meridional temperature gradients and intensifies wind speeds in the polar vortex. This mechanism could contribute through stratosphere-troposphere interaction to the generation of planetary waves, which can cause severe and long-term low temperatures during the winter in mid-latitudes^8^. A similar mechanism of systematic changes in precipitation due to solar radiation-induced cyclic changes in atmospheric pressure, is supported by studies of the Holocene’s annually laminated sediments in eastern Finland^134^. These inferences are strengthened by recent satellite observations that UV band variations between different phases of solar cycles are much more pronounced than previously thought^4^. In addition, the models which include this substantial variability in UV radiation successfully replicate the currently observed patterns of severe winter temperatures in the USA and northern Europe^149^. Observations of the amplitudes of perturbations during different phases of 11-year solar cycles have revealed that the strongest forcing is detectable in higher latitudes, which is most probably related to variations in the strength of the circumpolar vortex^26^.

Wavelet analyses have revealed that the temperature proxy – NMDS1 shows much more persistent bicentennial cycling than the biodiversity metric 1/q*. This can be explained by the fact that at the beginning of the Holocene, the regional species pool, after the glaciers had retreated, was still far from a long-term equilibrium. Therefore, in these conditions, high-magnitude environmental fluctuations had smaller relative effects on the low background biodiversity. On the other hand, when the biodiversity reached its long-term peak values during the Holocene thermal maximum, sharp climatic fluctuations were manifest in the biodiversity changes. Therefore, the multimillennial assembly of the regional species pool was probably the primary reason for these discordances.

In addition, wavelet analyses have revealed that a major episode of high-magnitude fluctuations in pollen diversity occurred during the period between 6,000 and 4,000 cal yr BP. This instability was associated with the low phase of the ≈3,760-year cycle. This pattern could be putatively linked to the 4.2 ka BP climate instability and dryness event, which was determined based on sedimentological, paleobotanical and archeological studies in the northern hemisphere^150–152^. This event had a complex spatial structure^153^ caused by the interaction of decadal to millennial scale factors including orbital forcing^154,155^. Therefore, this study confirms the idea that this episode was a multiscale phenomenon, when multimillennial cycles modulated the effects of shorter-term (centennial) biodiversity and climatic fluctuations.

## Conclusions

Analysis of Holocene pollen data from the Čepkeliai bog revealed a hierarchy of interacting climatic factors that drove vegetation change. On the longest time scale of the entire Holocene, a unimodal parabolic trend prevails with a very distinct period of the Holocene Thermal Maximum, which shows consistent stasis in the pollen community composition, as reflected by the NMDS1 scores, and the inferred community diversity, as revealed by the 1/q* index. Community composition and pollen community diversity show statistically significant periodic variations on three scales. The longest periodicities of ≈3,760 and ≈1,880 years were most probably harmonically related to each other. These long cycles are consistent with two explanations: they could have been either a) higher harmonics (6^th^ and 12^th^ accordingly) of the precession index; or, alternatively, b) they could have been related to the long-term solar activity quasiperiodic variability which was detected in a comparable scale range in the Holocene^7^. The shorter-term periodicities within the range of between 240 and 201 years were very close to the duration of the DeVries solar quasicycles with an average duration of ~208 years. The strongest centennial cycles lasting 176 years were detected in the NMDS1 scores. They most probably represented the subharmonics of the Gleissberg solar cycle. The proximate mechanisms that affected the plant community composition could have originated from two mechanisms: a) through changes in the flux of cosmic rays, which induces changes in cloud cover and later modulates the influence of the marine climate on central Europe; and b) due to changes in ozone production and the heating of the stratosphere due to changes in UV radiation throughout the solar cycle, which induces changes in the meridional circumpolar circulation at mid- to high latitudes.

## Supplementary information

Dataset 1

## Data Availability

The NMDS1 scores and 1/q* scores are available in the Supporting Information.
